# Big data: Some statistical issues

**DOI:** 10.1016/j.spl.2018.02.015

**Published:** 2018-05

**Authors:** D.R. Cox, Christiana Kartsonaki, Ruth H. Keogh

**Affiliations:** a Nuffield College, Oxford OX1 1NF, UK; b Medical Research Council Population Health Research Unit and Clinical Trial Service Unit and Epidemiological Studies Unit,Nuffield Department of Population Health, University of Oxford, Oxford OX3 7LF, UK; c Department of Medical Statistics, London School of Hygiene and Tropical Medicine, Keppel Street, London WC1E 7HT, UK

**Keywords:** Big data, Electronic health records, Epidemiology, Metrology, Precision

## Abstract

A broad review is given of the impact of big data on various aspects of investigation. There is some but not total emphasis on issues in epidemiological research.

## 1 Introduction

Over the last 125 years computational techniques have evolved from slide rule and log tables, through hand operated machines like the Brunsviga, to electric desk-top machines, and from them to modern computers, at first complex to use and limited in scope and then to the ever expanding modern ubiquitous version. The development of statistical technique and theory over that time has mirrored and been strongly influenced by that growth in computer power and availability.

Big data have been around a long time, for example in population censuses. In an engineering context, paper traces recorded such properties as the stress at various points in an aircraft wing during flight. In a manufacturing context, the mass per unit length of textile yarn was recorded. These examples produced very large amounts of data for visual inspection, but in the past suitable for quantitative analysis at most on a sampling basis. Three questions that characterize today’s big data are largely absent from these earlier contexts. In outline the questions are: Are the data relevant for the purpose of the investigation? Is the data quality adequate for its intended purpose? Is the detailed statistical analysis appropriate, in particular is the assessment of the precision of the conclusions seriously overoptimistic? Sometimes the first two aspects may be inverted: the data are available, for what are they useful? We comment on these issues largely, but not entirely, from an epidemiological perspective.

In an epidemiological context, large datasets with many individuals arise from routinely collected medical records, from cohorts assembled with a defined objective, and from registries of patients with specific conditions. Some large population-based studies are of mixed type, in that they are cohorts with a purpose-built baseline dataset augmented by linkage to routinely collected records or registries. Many aspects of study design and analysis are common to large and not-so-large sets of data but the achievement of high quality in large sets of data may be a particular challenge.

There are a number of conceptual aspects of a study all of which may have statistical implications. These are: Question formulation; Choice of study population; Study design; Metrology; Data collection; Monitoring and quality control; Data analysis; Presentation of conclusions; Interpretation. When big data are involved all of these may raise special features. Here we concentrate largely but not entirely on the aspects prior to data analysis.

## 2 Some types of study

In a health context there are several ways in which big data may arise. One is via routine collection in so-called electronic-health (‘e-health’) record databases. In the UK the Clinical Practice Research Database (CPRD) (Herrett et al., 2015) has data on over 11 million patients from over 600 general practices. Information is recorded on individuals receiving normal care and the resulting data are sometimes described as ‘found’ data. Because the information on a patient arises from visits to a doctor the amount of data per patient is itself informative.

Another source of big data is patient registries with information on individuals with a specific condition, such as the UK and US cystic fibrosis patient registries. Data are more specific to the condition in question and the acquisition may include elements of both ‘design’ and ‘observation’. For example, the US Cystic Fibrosis Foundation Patient Registry collects data from patients’ visits to their care team, both routine and not, via a standardized approach using a web-based portal (Knapp et al., 2016). The UK Cystic Fibrosis registry obtains data annually at a visit arranged specifically to acquire data (Taylor-Robinson et al., in press).

Other large datasetsarise from cohorts recruited and followed with specific questions in mind, although may later be expanded to enable other investigations. An example is the Million Women Study (The Million Women Study Collaborative Group, 1999), the original primary aim of which was to study the relationship between use of hormone replacement therapy and the risk of breast cancer, and the European Prospective Investigation into Cancer and Nutrition, which aimed to study associations between diet, lifestyle and environmental factors, and the incidence of chronic diseases.

Many large-scale studies are designed to answer a wide-ranging set of questions over a period, allowing for new questions to emerge over time, rather than to address one or two specific pre-specified issues. An example in another field is the massive investigations in particle physics at CERN; these focused initially on finding the Higgs boson (ATLAS collaboration, 2012) but by the time that issue was resolved other wide-ranging searches for “new physics” were in progress. Developments in astrophysics raise special issues. Examples in health include the China Kadoorie Biobank Chen et al. (2005), Chen et al. (2011) and the UK Biobank (Collins, 2012), which obtain information across many domains thus allowing wide-ranging investigation. Biological samples collected in these studies also enable generating new data on these cohorts, perhaps as part of a nested case-control or case-subcohort study (Keogh and Cox, 2014).

Many large datasets consist of several datasets, each collected separately, such as consortia in genetic epidemiology, for example CIMBA (Chenevix-Trench et al., 2007).

## 3 Coverage and representativeness

Large datasets may not represent the underlying population of interest and sheer largeness of a dataset clearly does not imply that population parameters, such as prevalences or absolute risks, can be estimated without bias. However, lack of representativeness is less of a concern when the focus is on estimation of relative associations, for example risk ratios. Studies may also be designed not to be representative of the underlying population but using stratified sampling to capture a wide variety of participant characteristics. Registries of rare diseases are more likely to be representative of the relevant population. For example, the UK Cystic Fibrosis Registry contains data on nearly all individuals with Cystic Fibrosis in the UK.

There is often some self-selection of participants of large cohort studies. Self-selection is not necessarily a problem for investigation of associations and dependences. However, biased estimates of associations of interest may arise if factors affecting selection are not accounted for by analysis, and especially if there are important factors affecting selection that are unknown or unmeasured and yet associated with the main factors under investigation.

A particular advantage of large datasetsis that they can cover a number of underlying subpopulations with particular features, e.g. age, ethnicity and socioeconomic status, which enable investigations of the stability of conclusions across different groups. The opt-out nature of electronic health databases such as the CPRD mean that patients with a wider range of characteristics are captured relative to studies in which participants opt in Herrett et al. (2015). Issues of representativeness arise also in sociological investigations, for example of the impact of parental social class on aspects of the childrens’ life (Goldthorpe, 2016). In such work formal probabilistically based sampling techniques are more likely to be employed but response rates are often so low that the formal justification of inference to the target population may be unconvincing. Instead justification of conclusions about the target population is, as in the epidemiological context, more firmly based on showing the stability of the conclusions, that is absence of statistical interaction with major features.

## 4 Metrology

Metrology, that is issues of measurement, is central to progress in many fields. ‘Big data’ often comprise data that are not just large in volume but also complex. Just a few instances in a biomedical context are data obtained from genotyping arrays, platforms for proteomics, metabolomics and transcriptomics, accelerometers to measure sleep and physical activity, devices that measure physical features, brain and other body scans, and geocoded air pollution data. New fields of study may be opened up by reliable methods of measurement becoming available for relatively routine use.

However elaborate the method or instrument employed some key principles remain, some of them inherited from the early work at national organizations such as the National Bureau of Standards in Washington DC, the National Physical Laboratory in the UK, and also by organizations specifically concerned with implementation of standards. Aspects of the monitoring of industrial quality and sampling inspection were intensively studied from the 1930s onwards and extended to such topics as the auditing of accounts by monetary unit sampling. The much more elaborate largely automated methods now in common use raise essentially the same issues of the standardization of different instruments and of checks of their stability and detection of occasional malfunction. If, as is sometimes the case, internal computation is involved this too may require monitoring. When, as in the interpretation of X-rays, an element of subjective judgement is involved research investigations may need randomization to ensure concealment and absence of measurement bias. It is unclear to what extent the expertise and experience of the earlier work has been continued and extended.

An important consideration is the design of questionnaires and interview-based instruments. Device data may be collected to replace self-reported information which may be less accurate and have potential for bias; for example sleep and physical activity data suggest that the amount of time spent in these states as perceived by the study individual may differ substantially from data measured using accelerometers. Another purpose is to collect several ‘layers’ of data on the same underlying process at various stages of the causal chain to gain more detailed insight on the process, for example genomic and downstream transcriptomic and proteomic data. Collecting such layers of data also opens new possibilities in the way that the data are used, for example in an instrumental variables approach. Genetic data are often used combined with epidemiological exposure data in Mendelian randomization analyses which help assess whether an observed association may reflect a potentially causal relationship, although the assumptions involved are not always easily checked.

In some cases error is introduced in the process of generating the data themselves. For the types of data discussed above complex pre-processing is required before analysis involving important decisions. For example, imaging data and accelerometer data may be pre-processed in various ways before different features are extracted to be used in downstream analysis. Spatial air pollution, temperature or humidity data may be assigned to individuals using different methods.

New ways of collecting data are sometimes used, such as data being collected by ‘lay’ persons. An example is ‘crowd sourcing’ where people help generate data; another situation is when individuals collect data about themselves, such as symptom monitoring, commonly using a phone application, which enables individuals to report their symptoms in their natural environment rather than in a doctor’s office and at more frequent times. There may be different issues related to the collection, quality check and analysis of such data.

## 5 Data quality

A sense that having data on a very large number of individuals renders problems of study design or data quality such as errors of measurement and missing data unimportant is usually misguided. One consequence of nominally small standard errors of estimation may be to make potential biases of more concern.

Routinely collected data may allow addressing many different questions. However, because the data are not collected for a specific purpose they may be subject to particular issues of data quality. For example, the information recorded in electronic health records by different doctors may vary in detail and accuracy. Data may be missing for a variety of reasons in such records, for reasons likely to depend on observed and unobserved patient characteristics. The CPRD encourages data quality via an incentive scheme (Herrett et al., 2015). The data recorded rely on measures used in routine care, which may not be the gold standard or the best for addressing a particular question. Greater control over some aspects of data collection and quality is possible in cohorts assembled for research. An example with high quality of data collection is the China Kadoorie Biobank, with data collection using streamlined procedures implemented by trained staff, built-in quality control checks, e.g. for implausible values, a quality control survey of a subset of participants, and regular monitoring of newly collected data. Outcome data obtained via linkage with health insurance records, disease and death registries are enhanced by reviews of residential records and local visits. Event adjudication is carried out for major outcomes to minimize misclassification and obtain more details. Statistical sampling inspection and quality control have long histories in industrial contexts starting from the early 1930s and extending into such fields as the auditing of accounts.

The availability of large numbers of variables in routinely collected data makes adjustment for large numbers of potential confounders possible; this brings its own challenges for analysis and special methods have been developed for adjusting for large numbers of confounders (Schneeweiss et al., 2009). Cohorts, such as population-based epidemiological studies designed to investigate a large number of exposures and outcomes, may not record very detailed information on all domains and may therefore lack detailed variables for some investigations needed either as exposures, outcomes or confounders.

Datasetswith health information are often linked to other data, such as from hospital episodes. Biases can be introduced in such linkage, in particular when the different databases do not contain the same unique individual identifiers (Harron et al., 2014).

When data are derived from device measurements, the data produced must be thoroughly checked for various types of error that may arise. In some specific fields there are well established quality control and assurance procedures, such as in genetic epidemiology.

## 6 Analysis and precision

Details of analysis will not be discussed in this paper. A broad strategic issue particularly prominent for big data is the contrast between analysing the data in relatively compact subsections aiming for an ultimate synthesis versus a one-step analysis involving the fitting of a relatively complex model. The former may be much slower but often more secure.

Conventional statistical thinking emphasizes, often over-emphasizes, the underpinning of statistical analysis by formal probability models. By contrast some approaches, such as neural nets for unravelling complex dependences, are solely algorithmic.

Most although not all relatively standard statistical procedures produce, after due precaution against anomalies, estimates with standard errors inversely proportional to the square root of sample size. For big data these standard errors are thus likely to be extremely small. Yet, to take just one example, the notion that the difference in mean survival times comparing two treatment regimens can be defined and estimated meaningfully with very high precision is unconvincing.

In fact there is evidence from many fields that when data are examined with a broad horizon standard errors may decrease inversely as a smaller power of sample size, for example as the one-quarter power. Examples include hydrology, the so-called Hurst effect based on studies of the River Nile, agriculture, error versus plot size, and turbulence. For the direct implications for statistical analysis, treated theoretically, see Cox (2016). Thus, whereas in some contexts big data may decrease the importance of precision assessment, in others special care may be needed.

There are also computational issues, in that when data are sufficiently complex and multiple steps of relatively standard processing is required, pre-specified schemes of analysis are often used. This may be useful in making analysis more efficient and reproducible and in minimizing human error. Sometimes, however, issues that arise in the middle of the process may be overlooked if there is not sufficient diagnostic information produced and sometimes inferior analyses may result.

There are also issues to be considered in the analysis of particular types of data. In electronic health records and in some patient registries, data are recorded each time the patient has contact with their health care system. A consequence of this is that data are recorded more frequently for patients with poorer health. Informative observation of participants presents challenges for statistical analyses making use of longitudinal measurements, and could result in biased inferences if not handled correctly.

Another particular situation has a very large number of variables of the same type, such as genomic variables, possibly on a relatively small number of individuals, in which if variables are considered simultaneously, interpretation relies on an assumed sparsity of effects Tibshirani (1996), Cox and Battey (2017).

## 7 Concluding remarks

Big data enable investigations to be conducted and reliable conclusions to be drawn that would otherwise be difficult or impossible. An example is their use in pharmacoepidemiology to evaluate treatment effects Smeeth et al. (2009), Hernan and Robins (2016).

We have highlighted some of the challenges that arise in the use of big data. One main theme has been to emphasize the potential for overconfidence in results obtained from analyses of large datasets,due to superficially highly precise but potentially biased estimates, or due to under-estimated standard errors. The size of the data does not remove the need for appropriate study design and statistical analysis (e.g. Welch et al., 2014, Lin et al., 2004, Pullenayegum and Lim, 2016). The potential impact of unobserved and unaccounted-for dependencies must not be ignored.

We have focused primarily on big data in the biomedical field. Even within that field there are many sources of big data that we have not mentioned. Each may present special challenges and opportunities.

In summary, while the availability of big data offers many possibilities for improved understanding, the need for careful and productive use of statistical concepts is pervasive and raises many challenges.
